# Positional Respiratory Distress in Myasthenic Crisis: A Case of Undiagnosed Thymoma

**DOI:** 10.7759/cureus.99322

**Published:** 2025-12-15

**Authors:** Oscar Diaz, Noah M Krupnick, Yadaymis Hidalgo Jimenez, Lazaro Basart, Benjamin Graham, Evelin P Jimenez

**Affiliations:** 1 Internal Medicine, Palmetto General Hospital, Hialeah, USA; 2 Internal Medicine, Dr. Kiran C. Patel College of Osteopathic Medicine, Nova Southeastern University, Fort Lauderdale, USA; 3 Pathology, Palmetto General Hospital, Hialeah, USA

**Keywords:** autoimmune disorder, myasthenia gravis (mg), myasthenic crisis, respiratory compromise, thymoma

## Abstract

Myasthenia gravis (MG) is a chronic autoimmune neuromuscular disorder that can present with life-threatening complications, including myasthenic crisis (MC). We present the case of a 52-year-old Hispanic male with known MG who presented to a hospital serving an underserved community with respiratory distress after discontinuing azathioprine. He was initially presumed to be experiencing a typical MC but then exhibited positional respiratory compromise, an inability to tolerate the supine position and the inability to have a nasogastric tube placed, which raised concern for dynamic extrinsic compression of mediastinal structures. These atypical findings prompted imaging, which revealed a large anterior mediastinal mass consistent with thymoma. The patient’s respiratory status worsened when supine, suggesting posture-dependent mass effect likely exacerbated by gravitational forces and impaired diaphragmatic mechanics. The tumor was found to be abutting both phrenic nerves, contributing to diaphragmatic dysfunction and worsening bulbar symptoms. He underwent successful surgical resection but required prolonged postoperative support, including tracheostomy and gastrostomy tube placement. This case highlights the need for heightened clinical suspicion of thymoma in MG patients presenting with crisis, particularly when positional symptoms suggest dynamic mass effect, as early recognition and intervention are essential for optimizing outcomes.

## Introduction

Myasthenia gravis (MG) is a chronic autoimmune neuromuscular disorder characterized by fluctuating and fatigable muscle weakness, most commonly affecting ocular, bulbar, limb, and respiratory functions. The condition is commonly associated with autoantibodies directed against acetylcholine receptors at the neuromuscular junction, impairing synaptic transmission and leading to activity-dependent muscle weakness [1]. Management may include acetylcholinesterase inhibitors, immunotherapy, immunomodulation, monoclonal antibodies, and thymectomy [2].

MG is the most common disorder of the neuromuscular junction and is associated with significant morbidity and mortality. Its global incidence has been rising, likely due to improved diagnostic accuracy, increased clinical awareness, and longer life expectancy. The current prevalence is estimated at 150 to 200 cases per million individuals. Although the overall mortality rate has declined significantly over the past century due to advances in medical therapy, outcomes remain worse in patients who experience myasthenic crisis (MC) [3].

MC is the most severe complication of MG and occurs in 15-20% of patients. It is characterized by severe weakness of respiratory and upper airway muscles, necessitating mechanical ventilation. It may represent the initial presentation of the disease or be related to disease exacerbation. Common triggers of MC include infections, medication adjustments, and medication non-adherence [4,5]. The mortality rate for patients experiencing MC is approximately 5-12% [6].

Thymomas are identified in approximately one-third of MC patients [7]. These are rare epithelial tumors of the anterior mediastinum and may be asymptomatic in up to 30% of cases. When symptoms occur, they are often due to mass effect or local invasion of adjacent structures such as the trachea, esophagus, recurrent laryngeal nerve, phrenic nerves, or superior vena cava. The most commonly reported symptoms include cough, chest pain, and dyspnea. Infiltration of the superior vena cava may result in superior vena cava syndrome, while involvement of the phrenic nerves can lead to diaphragmatic paralysis. Compression of the trachea or esophagus may also manifest as stridor or dysphagia, respectively [8-10].

Thymomas may also present with paraneoplastic syndromes. Among these, MG is the most common [8,9]. Up to 15% of individuals with MG have an associated thymoma, while approximately 30-40% of patients with thymoma develop symptoms of MG [2]. The presence of thymoma increases the risk of developing MC [5]. MG associated with thymoma is typically more severe and is often associated with a poorer prognosis compared to non-thymomatous MG [2,11,12]. Thymectomy is generally indicated in patients with MG and confirmed thymoma. It has also been shown to increase the likelihood of overall improvement in cases of non-thymomatous, antibody-positive MG [2].

This report presents the case of a Hispanic male with MG who was admitted in MC to a tertiary care center serving an underserved population, where imaging revealed a previously undiagnosed thymoma. The case underscores the importance of maintaining a high index of suspicion for thymoma in patients with MG, particularly in medically underserved populations where delayed diagnosis may contribute to more severe clinical presentations.

## Case presentation

A 52-year-old Hispanic male with a known history of MG, but no other significant medical history, presented to our hospital, which provides care to a medically underserved community, with two weeks of progressively worsening generalized weakness, dysphagia, and dyspnea, which had significantly intensified the morning of admission. He reported mild exertional dyspnea but denied fever, chills, cough, or chest pain. He had been receiving outpatient care from a neurologist at an outside institution and was previously maintained on azathioprine, prednisone, and pyridostigmine. Notably, he had recently discontinued azathioprine for unclear reasons but had continued prednisone (5 mg PO daily) and pyridostigmine (180 mg every six hours).

On presentation, his vital signs were within normal limits, aside from a room air oxygen saturation of 94%. He was alert, oriented, and in mild respiratory distress. Physical examination revealed generalized muscle weakness and bilateral ptosis. There were no focal neurological deficits. No fasciculations or tongue atrophy was observed. Lungs were clear to auscultation, and the cardiac exam was unremarkable, with no murmurs, gallops, or rubs.

**Figure 1:**
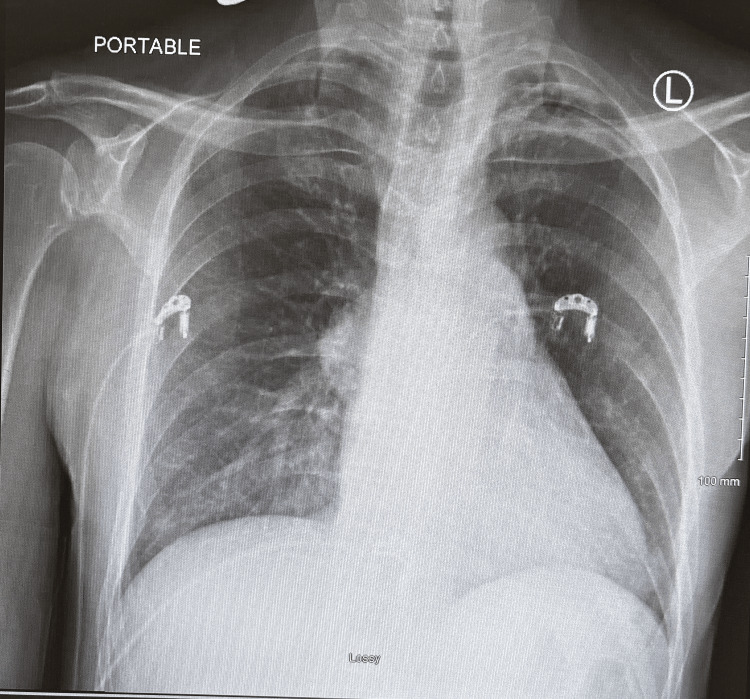
Chest X-ray from admission showing no acute cardiopulmonary pathology

Initial laboratory results are shown in Table 1, notable for mild leukocytosis. Acetylcholine receptor blocking, binding, and modulating antibodies were all positive. No other serum MG markers were tested. Radiology reading of the admission chest X-ray reported no acute cardiopulmonary pathology (Figure 1).

**Table 1:** Laboratory values on admission WBC, white blood cells; RBC, red blood cells; Hgb, hemoglobin; Hct, hematocrit; MCV, mean corpuscular volume; PT, prothrombin time; INR, international normalized ratio; aPTT, activated partial thromboplastin time; pCO_2_, arterial blood partial pressure CO_2_; pO_2_, arterial blood partial pressure O_2_; HCO_3_, arterial blood HCO_3_

Test	Patient Value	Normal
Complete blood count		
WBC	12.1 x 10^3^/uL	5.0-11.0 x 10^3^/uL
RBC	4.89 x 10^6^/mm^3^	4.70-6.10 x 10^6^/mm^3^
Hgb	15.1 gm/dL	14.0-18.0 gm/dL
Hct	44.8%	42-52.0%
MCV	92 fL	80-94 fL
PT	10.8 seconds	9.4-12.5 seconds
INR	1.0	0.9-1.2
aPTT	24.9	20-38.0 seconds
Arterial blood gas		
pH	7.44	7.35-7.45
pCO₂	39 mmHg	35.0-45.0 mmHg
pO₂	85.6 mmHg	75.00-100.00 mmHg
HCO_3_	26 mmol/L	22-26 mmol/L
Complete metabolic panel		
Sodium	142 mmol/L	137-145 mmol/L
Potassium	4.5 mmol/L	3.4-5.0 mmol/L
Chloride	110 mmol/L	98-107 mmol/L
Carbon dioxide	30mmol/L	22-30 mmol/L

The patient was admitted to the intensive care unit (ICU) for the management of MC, with an initial treatment plan consisting of a seven-day course of daily plasmapheresis, high-dose corticosteroids (methylprednisolone 30 mg IV every six hours), and intravenous immunoglobulin (400 mg/kg over 5 days). Pyridostigmine was discontinued on admission. Several hours after admission, he developed difficulty clearing oral secretions. Placement in the supine position was required to establish adequate central venous access for plasmapheresis, but this resulted in marked respiratory decompensation. Despite trial of bilevel positive airway pressure (BiPAP) and mild sedation, the patient could not tolerate lying supine and subsequently required endotracheal intubation. Post-intubation arterial blood gas revealed significant hypercapnia and acidosis (Table 2).

**Table 2:** Repeat arterial blood gas pCO_2,_ arterial blood partial pressure CO_2_; pO_2_, arterial blood partial pressure O_2_; HCO_3_, arterial blood HCO_3_

Value Name	Patient value	Normal value
Arterial blood gas		
pH	7.08	7.35-7.45
pCO₂	64.6 mmHg	35.0-45.0 mmHg
pO₂	555.8 mmHg	75.00-100.00 mmHg
HCO_3_	19 mmol/L	22-26 mmol/L

**Figure 2:**
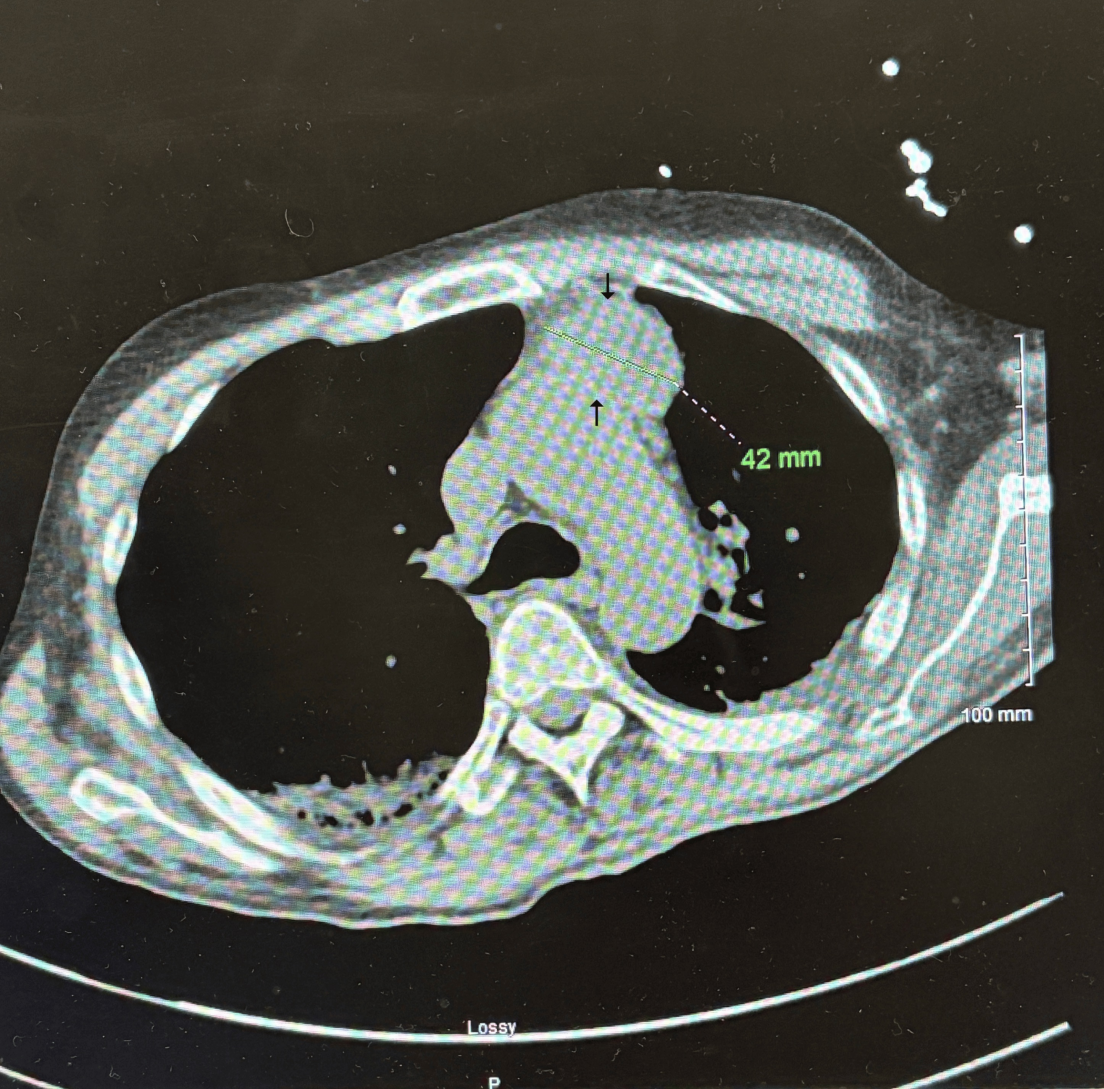
Chest CT revealing a large anterior mediastinal mass

Repeated unsuccessful attempts at nasogastric (NG) tube placement raised concern for a mechanical obstruction or mass lesion. A CT scan of the chest was reported as demonstrating a well-defined, lobulated, soft tissue mass in the anterior mediastinum, measuring approximately 4.2 cm in transverse diameter. There was no evidence of gross invasion into adjacent mediastinal structures (Figure 2).

**Figure 3:**
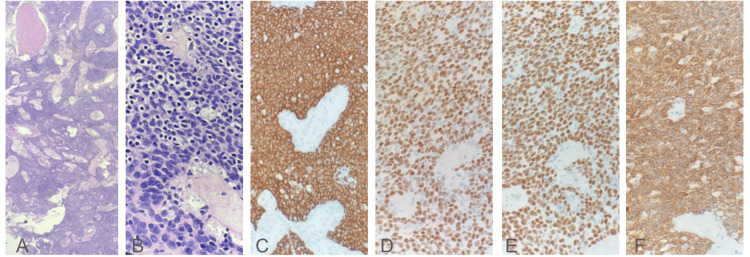
Histopathology and immunohistochemistry images of thymic mass. A. Low-power view of WHO type B2 thymoma shows increased epithelioid-appearing neoplastic thymic epithelial cells B. High-power view of WHO type B2 thymoma shows increased epithelioid-appearing neoplastic thymic epithelial cells with admixed small round blue lymphocytes C. CK5/6 immunohistochemical stain highlighting epithelioid-appearing neoplastic thymic epithelial cells D. P63 immunohistochemical stain highlighting epithelioid-appearing neoplastic thymic epithelial cells E. P40 immunohistochemical stain highlighting epithelioid-appearing neoplastic thymic epithelial cells F. Pankeratin immunohistochemical stain highlighting epithelioid-appearing neoplastic thymic epithelial cells

The patient was evaluated by cardiothoracic surgery and subsequently underwent median sternotomy with complete resection of the mass. The mass was noted to be abutting both phrenic nerves, which were successfully dissected free. Pathologic evaluation revealed an encapsulated mass measuring 8.5 × 8 × 3 cm and weighing 85 grams. Pathology report confirmed a thymoma, WHO type B2. Immunohistochemical staining revealed neoplastic cells positive for CK5/6, pankeratin, p40, and p63, (Figure 3) and negative for CD117 and CK7. 

Postoperatively, the patient was returned to the ICU for continued monitoring. His recovery was complicated by persistent difficulty with extubation due to copious secretions. A therapeutic bronchoscopy was performed, and respiratory cultures grew Proteus species, which was treated with intravenous ceftriaxone. Given his ongoing need for airway protection and enteral nutrition, tracheostomy and percutaneous endoscopic gastrostomy (PEG) tubes were placed. He was weaned off the high-dose steroids and restarted on pyridostigmine 60 mg per gastrostomy tube thrice a day and was ultimately transferred to a long-term acute care facility for continued recovery and rehabilitation.

## Discussion

This case highlights the importance of maintaining a high index of suspicion for thymoma in all patients with MG, particularly those who present in crisis. Our patient presented with MC, possibly precipitated by recent discontinuation of azathioprine, a commonly used steroid-sparing immunosuppressive agent. Reduction or cessation of immunosuppressive therapy is a well-recognized trigger for MC [1,4]. In one study, 53% of patients had reactivation of disease less than one year after discontinuation of azathioprine [13]. Azathioprine, while often first-line agent, is more frequently discontinued than other agents due to its adverse effects [14].

Initially, the patient’s clinical deterioration was attributed to MC. His pyridostigmine was stopped, as its potential benefit in MC is outweighed by its risk of promoting secretions and mucus plugging [7]. However, the exacerbation of his respiratory distress while supine and inability to pass an NG tube are not typical of MC alone. and this raised concern for an additional pathologic process. These positional symptoms can be observed with large anterior mediastinal masses, which can exert a compressive effect on mediastinal structures, particularly in the supine position [15]. In this position, gravitational forces can exacerbate compression of mediastinal structures, leading to respiratory compromise, dysphagia, or mechanical compression impairing NG tube placement [16]. Additionally, cephalad displacement of the diaphragm while lying supine reduces thoracic volume, limiting the space available for the trachea relative to the mass, and further compromises respiratory function [16,17]. This effect can be amplified in patients with weakened diaphragmatic or intercostal muscles [16], as seen in MG. Imaging revealed a previously undiagnosed anterior mediastinal mass, later confirmed to be a thymoma. It is notable that our patient was positive for acetylcholine receptor antibodies. While these antibodies are present in about 85% of individuals with MG, they are seen in almost all patients with thymomas [18].

In our patient, the thymoma was adherent to the phrenic nerves, which may have further compromised diaphragmatic function. Diaphragmatic impairment disrupts the normal coordination between respiration and swallowing, increasing the risk of ineffective swallowing and aspiration. In patients with MG, who are already predisposed to bulbar dysfunction, this added respiratory compromise can significantly worsen symptoms such as dysphagia and increase the likelihood of respiratory failure, and thus early recognition is critical.

## Conclusions

This case illustrates the complex interplay between MG, MC, and thymoma, and underscores the importance of considering structural causes of respiratory compromise in MG patients whose clinical picture deviates from a typical crisis. In this patient, the unexpected worsening of respiratory status and pooling of secretions in the supine position, as well as the difficulty with NG tube placement, were critical diagnostic clues, ultimately leading to the identification of a large anterior mediastinal mass. The mass was later confirmed to be a thymoma abutting both phrenic nerves, which likely contributed to respiratory insufficiency and dysphagia through impaired diaphragmatic function.

Early recognition and surgical management of thymoma are essential to optimize outcomes, particularly in MG patients presenting with atypical or severe features. This case reinforces the need for thorough evaluation in patients with MG who present with crisis, especially when symptoms such as postural respiratory distress or unexplained bulbar dysfunction are present. Clinicians should maintain a high index of suspicion for thymoma in such scenarios, particularly in medically underserved populations where delayed diagnosis may contribute to more severe clinical presentations, as timely diagnosis and intervention can significantly influence both short-term and long-term outcomes.
